# Equation of state and self-bound droplet in Rabi-coupled Bose mixtures

**DOI:** 10.1038/s41598-017-13647-y

**Published:** 2017-10-17

**Authors:** Alberto Cappellaro, Tommaso Macrì, Giovanni F. Bertacco, Luca Salasnich

**Affiliations:** 1 Dipartimento di Fisica e Astronomia “Galileo Galilei”, Università di Padova, via Marzolo 8, 35131 Padova, Italy; 2 Departamento de Física Teorica e Experimental, Universidade Federal do Rio Grande do Norte, and International Institute of Physics, 59070-405 Natal-RN, Brazil; 3 Istituto Nazionale di Ottica (INO) del Consiglio Nazionale delle Ricerche (CNR), via Nello Carrara 1, 50019 Sesto Fiorentino, Italy

## Abstract

Laser induced transitions between internal states of atoms have been playing a fundamental role to manipulate atomic clouds for many decades. In absence of interactions each atom behaves independently and their coherent quantum dynamics is described by the Rabi model. Since the experimental observation of Bose condensation in dilute gases, static and dynamical properties of multicomponent quantum gases have been extensively investigated. Moreover, at very low temperatures quantum fluctuations crucially affect the equation of state of many-body systems. Here we study the effects of quantum fluctuations on a Rabi-coupled two-component Bose gas of interacting alkali atoms. The divergent zero-point energy of gapless and gapped elementary excitations of the uniform system is properly regularized obtaining a meaningful analytical expression for the beyond-mean-field equation of state. In the case of attractive inter-particle interaction we show that the quantum pressure arising from Gaussian fluctuations can prevent the collapse of the mixture with the creation of a self-bound droplet. We characterize the droplet phase and discover an energetic instability above a critical Rabi frequency provoking the evaporation of the droplet. Finally, we suggest an experiment to observe such quantum droplets using Rabi-coupled internal states of K^39^ atoms.

## Introduction

In atomic physics, laser beams can stimulate transitions among different hyperfine states. Inspired by remarkable experiments with both fermionic^1,2^ and bosonic clouds^3,4^, in the recent years an extensive theoretical research was devoted to understand static and dynamical properties of quantum mixtures with artificial coupling beween their internal states. Concerning fermionic mixtures, for example, in the attempt to search for itinerant ferromagnetism driven by Rabi coupling, it was shown that a critical coupling frequency marks the transition of a two-state Fermi gas to a ferromagnetic phase. A detailed investigation in three spatial dimension was performed by Conduit^5^ and, very recently, for a two-dimensional Fermi gas^6^. On the other side, for bosonic atoms at temperatures below the transition to the superfluid phase, coupling of hyperfine states offers the possibility to address fascinating phenomena such as the internal Josephson effect^7–9^ emulating a space dependent double well potential, analogues of the Hawking radiation^10,11^, non-abelian gauge potentials^12^ like magnetic monopoles^13,14^, Rashba spin-orbit coupling^15–18^, or they can be used for applications to quantum metrology^19–21^ and for the quantum simulation of spin models with short or long-range interactions^22–25^.

In this article we study the effects of a Rabi coupling on a two-component Bose mixture deriving the corresponding beyond-mean-field equation of state. To achieve this result we perform a non-trivial regularization of Gaussian fluctuations, which have a divergent zero-point energy due to both gapless and gapped elementary excitations. In particular, we obtain a meaningful analytical formula for the ground-state energy of the Bose mixture as a function of Rabi coupling and scattering lengths. Setting the Rabi frequency to zero in our formula one recovers Larsen’s equation of state^26^. In the case of attractive inter-particle interaction we investigate the conditions for the formation of a self-bound droplet finding that its density profile and collective oscillations crucially depend on the interplay between Rabi coupling and interaction strengths. A similar equation of state, albeit in absence of internal coupling, has been recently used by Petrov^27,28^. He shows that, in the case of negative inter-component scattering length, quantum fluctuations can arrest the collapse of the mixture inducing the formation of a stable self-bound droplet. In a different context, the stabilization induced by quantum fluctuations has been found also in dipolar Bose-Einstein condensate, both in trapped configuration^29,30^ and in free space^31–34^.

Remarkably, we find that above a critical Rabi frequency the self-bound droplet evaporates into a uniform configuration of zero density. Finally, we analyze the most favorable conditions to obtain a stable self-bound droplet made of ^39^K atoms in two Rabi-coupled hyperfine states.

## Results

### Microscopic theory for Rabi-coupled mixtures

We consider a Bose gas with two relevant hyperfine states in a volume ${L}^{3}$, at temperature *T* and with chemical potential $\mu$. In addition to the usual intra- and inter-state contact interactions, transitions between the two states are induced by an an external coherent Rabi coupling of frequency ${\omega }_{R}$. We adopt the path integral formalism, where each component is described by a complex bosonic field ${{\rm{\psi }}}_{i}$ ($i=\mathrm{1,2}$). Given the spinor ${\boldsymbol{\Psi }}={({\psi }_{1},{\psi }_{2})}^{T}$ ^35–37^, the partition function of the system reads:

1 $${\mathscr{Z}}=\int {\mathscr{D}}[{\rm{\Psi }},\bar{{\rm{\Psi }}}]\exp (-\frac{1}{\hslash }S[{\rm{\Psi }},\bar{{\rm{\Psi }}}])\,,$$

where the Euclidean action $S[{\rm{\Psi }},\bar{{\rm{\Psi }}}]$ is given by

2 $$\begin{array}{rcl}S[{\rm{\Psi }},\bar{{\rm{\Psi }}}] & = & {\int }_{0}^{\beta \hslash }d\tau {\int }_{{L}^{3}}{d}^{3}{\bf{r}}\{\sum _{i\mathrm{=1,2}}[{\psi }_{i}^{\ast }(\hslash {\partial }_{\tau }-\frac{{\hslash }^{2}}{2m}{\nabla }^{2}-\mu ){\psi }_{i}+\frac{1}{2}{g}_{ii}|{\psi }_{i}{|}^{4}]\\  &  & +{g}_{12}|{\psi }_{1}{|}^{2}|{\psi }_{2}{|}^{2}-\hslash {\omega }_{R}({\psi }_{1}^{\ast }{\psi }_{2}+{\psi }_{2}^{\ast }{\psi }_{1})\},\end{array}$$

with $\beta \equiv 1/({k}_{B}T)$ and ${g}_{ij}=4\pi {\hslash }^{2}{a}_{ij}/m$ being ${a}_{ij}$ the scattering length for collisions between component *i* and component *j* (specifically *a*_11_, *a*_22_, and *a*_12_). All relevant thermodynamical quantities can be derived from the grand potential ${\rm{\Omega }}=-\frac{1}{\beta }\,{\rm{l}}{\rm{n}}({\mathscr{Z}})$. We work in the superfluid phase, where a U(1) gauge symmetry of each bosonic component is spontaneously broken. The presence of the Rabi coupling in the Euclidean action in equation (2) implies that only the total number of atoms is conserved. We can then set ${\psi }_{i}({\bf{r}},\tau )={v}_{i}+{\eta }_{i}({\bf{r}},\tau )$, where *v*_*i*_ are the uniform order parameters of the two-component Bose-Einstein condensate, and ${\eta }_{i}(r,\tau )$ are the fluctuation fields above the condensate. The mean-field plus gaussian approximation is obtained by expanding equation (2) up to the second order in ${\eta }_{i}({\rm{r}},\tau )$ and ${\eta }_{i}^{\ast }({\rm{r}},\tau )$. The corresponding beyond-mean-field grand potential is then given by^37,38^

3 $${\rm{\Omega }}(\mu ,{v}_{1},{v}_{2})={{\rm{\Omega }}}_{0}(\mu ,{v}_{1},{v}_{2})+{{\rm{\Omega }}}_{g}(\mu ,{v}_{1},{v}_{2}),$$

where

4 $$\begin{array}{l}{{\rm{\Omega }}}_{0}(\mu ,{v}_{1},{v}_{2})={L}^{3}[\sum _{i\mathrm{=1,2}}(-\mu {v}_{i}^{2}+\frac{1}{2}{g}_{ii}{v}_{i}^{4})+{g}_{12}{v}_{1}^{2}{v}_{2}^{2}-2\hslash {\omega }_{R}{v}_{1}{v}_{2}]\end{array}$$

is the mean-field grand potential, while ${{\rm{\Omega }}}_{g}(\mu ,{v}_{1},{v}_{2})$ is the grand potential of Gaussian quantum and thermal fluctuations.

**Figure 1:**
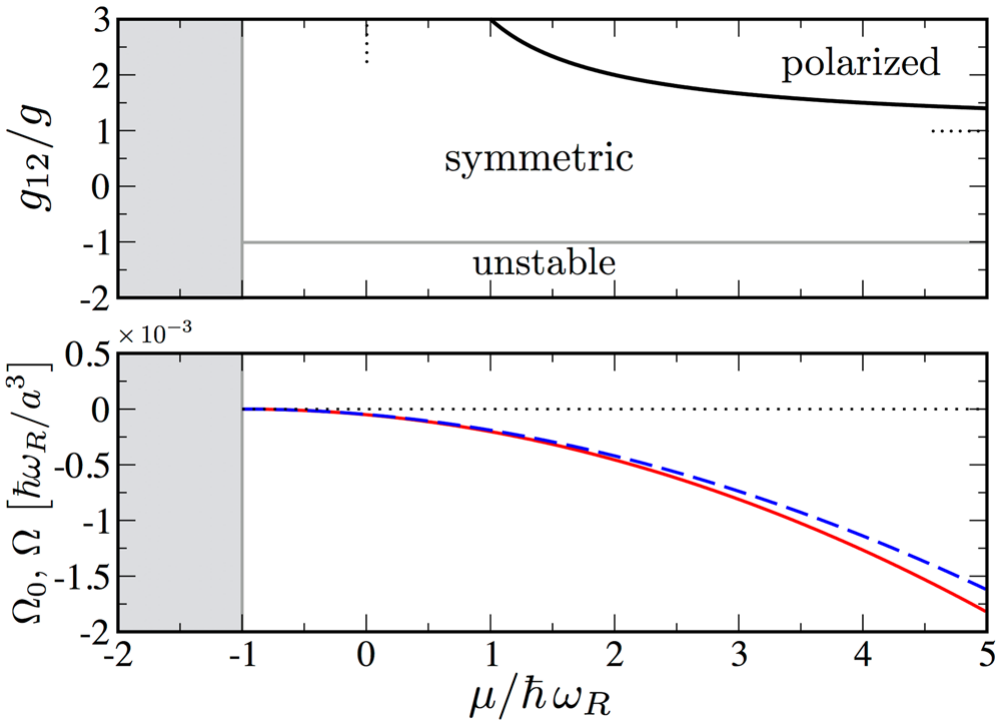
Grand canonical phase diagram and grand potential. (Top) Mean field phase diagram based on the grand potential ${{\rm{\Omega }}}_{0}(\mu ,{\nu }_{1},{\nu }_{2})$ of equation (4). In the symmetric ground state the two components appear with the same particle density $|{\nu }_{1}{|}^{2}=|{\nu }_{2}{|}^{2}$, whereas in the polarized phase densities are unequal. Dotted lines represent the asymptotic phase boundaries of the polarized region for large ${g}_{12}/g$ and $\mu /\hslash \,{\omega }_{R}$ ratios respectively. For ${g}_{12}/g\, < \,-1$ the symmetric solution is unstable in the *thermodynamic limit*. The grey region for $\mu  < \hslash \,{\omega }_{R}$ corresponds to the trivial solution $|{\nu }_{1}{|}^{2}=|{\nu }_{2}{|}^{2}=0$. (Bottom) Grand potential ${\rm{\Omega }}(\mu )$ (dashed blue line) with the inclusion of gaussian fluctuations of equation (12) and its mean field approximation ${{\rm{\Omega }}}_{0}(\mu )$ (red solid line) of equation (7) as a function of the chemical potential $\mu$ for ${g}_{12}=0.9g$ within the symmetric phase.

In our scheme, the Bose-Einstein order parameters ${v}_{i}$ satisfy the saddle-point equations $\partial {{\rm{\Omega }}}_{0}(\mu ,{v}_{1},{v}_{2})/\partial {v}_{i}=0$, leading to coupled equations for the uniform and constant fields ${v}_{1}$ and ${v}_{2}$:

5 $$({g}_{ii}{v}_{i}^{2}+{g}_{ij}{v}_{j}^{2}){v}_{i}-\hslash {\omega }_{R}{v}_{j}=\mu {v}_{i}$$

with $i=\mathrm{1,}\,2$ and $j\ne i$. The analysis of the minima of ${{\rm{\Omega }}}_{0}(\mu ,{v}_{1},{v}_{2})$ at the solution of equations (5) leads to the mean field phase diagram of Fig. 1 (top panel) which is obtained for the case of equal intra-component *repulsive* interaction strength ${g}_{11}={g}_{22}\equiv g$.

One finds a symmetric configuration where the two internal states are equally populated, a polarized phase with non-zero population imbalance, and an unstable phase when the attractive inter-state interaction overcomes the intra-state repulsion ${g}_{12} < \,-g$ ^39–42^ (see Methods for some technical details).

In the rest of this article we focus on the symmetric ground state existing in presence of Rabi coupling, where ${v}_{1}={v}_{2}\equiv v/\sqrt{2}$ and equal intra-component interaction. The corresponding mean-field grand potential ${{\rm{\Omega }}}_{0}(\mu ,v)$ is then given by

6 $$\frac{{{\rm{\Omega }}}_{0}(\mu ,v)}{{L}^{3}}=-\mu {v}^{2}+\frac{1}{4}(g+{g}_{12}){v}^{4}-\hslash {\omega }_{R}{v}^{2}\mathrm{.}$$

By solving equation (5) in the case of symmetric ground-state, we get the crucial relation between the order parameter and the chemical potential: ${v}^{2}=\mathrm{2(}\mu +\hslash {\omega }_{R})/(g+{g}_{12})$. In this case, equation (6) reduces to

7 $$\frac{{{\rm{\Omega }}}_{0}(\mu )}{{L}^{3}}=-\frac{{(\mu +\hslash {\omega }_{R})}^{2}}{g+{g}_{12}}\mathrm{.}$$

It is important to stress that we work in a regime where Rabi coupling cannot produce polarization in the ground state. However, as shown in the following section, it still influences the stability of balanced configuration, i.e. the region between the symmetric and unstable phase in the diagram reported in Fig. 1 (top panel), also when Gaussian fluctuations are taken into account.

### Gaussian Fluctuations

To compute ${{\rm{\Omega }}}_{g}(\mu ,v)$ for the symmetric ground state and for equal interaction strengths, we consider the quadratic terms in ${\eta }_{i}$ and ${\eta }_{i}^{\ast }$ of equation (2). In reciprocal Fourier space one finds

8 $${S}_{2}[{\boldsymbol{\eta }}({\bf{q}},{\omega }_{n}),\bar{{\boldsymbol{\eta }}}({\bf{q}},{\omega }_{n})]=-\frac{\hslash }{2}\sum _{{\bf{q}},{\omega }_{n}}\bar{{\boldsymbol{\eta }}}({\bf{q}},{\omega }_{n}){\mathbb{M}}({\bf{q}},{\omega }_{n})\,{\boldsymbol{\eta }}({\bf{q}},{\omega }_{n}\mathrm{).}$$

Here ${\{{\omega }_{n}\}}_{n}$ are the bosonic Matsubara frequencies and $-\hslash {\mathbb{M}}({\bf{q}},{\omega }_{n})$ is the $4\times 4$ inverse of the fluctuations propagator, whose definition is reported in the Methods. At zero temperature, the Gaussian grand potential corresponds to the zero-point energy of bosonic excitations and it reads^37,43^

9 $${{\rm{\Omega }}}_{g}(\mu ,v)=\frac{1}{2}\sum _{{\bf{q}}}[{E}_{{\bf{q}}}^{(+)}(\mu ,v)+{E}_{{\bf{q}}}^{(-)}(\mu ,v)],$$

where ${E}_{{\bf{q}}}^{(\pm )}(\mu ,v)$ is the spectrum of elementary excitations, which can be obtained by diagonalizing $-\hslash [{\mathbb{I}}\cdot {\mathbb{M}}({\bf{q}}\mathrm{,0)]}$ ^35,37,44^. The diagonal blocks of ${\mathbb{I}}$ are two-by-two identity matrices ${{\bf{1}}}_{2}$, while the off-diagonal ones are the Pauli matrix ${\sigma }_{z}$. The eigenvalues are the two branches of the Bogoliubov spectrum:

10 $${E}_{{\bf{q}}}^{(+)}=\sqrt{\frac{{\hslash }^{2}{q}^{2}}{2m}[\frac{{\hslash }^{2}{q}^{2}}{2m}+\mathrm{2(}\mu +\hslash {\omega }_{R})]}$$

and

11 $${E}_{{\bf{q}}}^{(-)}=\sqrt{\frac{{\hslash }^{2}{q}^{2}}{2m}[\frac{{\hslash }^{2}{q}^{2}}{2m}+2A(\mu ,{\omega }_{R})]+B(\mu ,{\omega }_{R})},$$

where we set $\varepsilon ={a}_{12}/a$, with $a$ the intra-component scattering length and ${a}_{12}$ the inter-component scattering lengt, $A=(\mu +\hslash {\omega }_{R}\mathrm{)(1}-\varepsilon )/\mathrm{(1}+\varepsilon )+2\hslash {\omega }_{R}$ and $B=4\hslash {\omega }_{R}[(\mu +\hslash {\omega }_{R}\mathrm{)(1}-\varepsilon )/\mathrm{(1}+\varepsilon )+\hslash {\omega }_{R}]\mathrm{.}$ In the continuum limit ${\sum }_{{\bf{q}}}\to {L}^{3}\int {d}^{3}{\bf{q}}/{\mathrm{(2}\pi )}^{3}$, the zero-temperature Gaussian grand potential is ultraviolet divergent. We employ the convergence-factor regularization^37,43,45^ which generates proper counterterms in the zero-point energy completely removing the divergence. These counterterms can be determined by expanding the two branches of the Bogoliubov spectrum at high momenta. The zero-temperature beyond-mean-field grand potential is then given by equation (7) plus the regularized zero-point energy, namely

12 $$\begin{array}{ll}\frac{{\rm{\Omega }}(\mu )}{{L}^{3}} & =-\frac{{(\mu +\hslash {\omega }_{R})}^{2}}{g+{g}_{12}}+\frac{8}{15{\pi }^{2}}{(\frac{m}{{\hslash }^{2}})}^{\mathrm{3/2}}{(\mu +\hslash {\omega }_{R})}^{\mathrm{5/2}}+\frac{{A}^{\mathrm{5/2}}}{2\sqrt{2}{\pi }^{2}}{(\frac{m}{{\hslash }^{2}})}^{\mathrm{3/2}}I(\mu ,{\omega }_{R},\varepsilon \mathrm{).}\end{array}$$

The function $I(\mu ,{\omega }_{R},\varepsilon )$ is given by

13 $$I(\mu ,{\omega }_{R},\varepsilon )={\int }_{0}^{+\infty }dy[\sqrt{y}\sqrt{{y}^{2}+2y+\frac{B}{{A}^{2}}}-{y}^{\mathrm{3/2}}-\sqrt{y}-\frac{(\frac{B}{{A}^{2}}-1)}{2\sqrt{y}}].$$

In Fig. 1 (bottom panel) we plot the grand potential ${\rm{\Omega }}(\mu )$ of equation (12), including gaussian fluctuations, as a function of the chemical potential for ${g}_{12}=0.9g$. We compare it with the mean field approximation ${{\rm{\Omega }}}_{0}(\mu )$ of equation (7). The energy density of the system is ${\mathcal E} =E/{L}^{3}={\rm{\Omega }}/{L}^{3}+\mu n$ where the number density *n* is obtained via $n=-\frac{1}{{L}^{3}}\frac{\partial {\rm{\Omega }}}{\partial \mu }$. In the limit of small Rabi-coupling, which is also the most relevant experimentally^10^ (see below), it is possible to get an analytical result for the energy density. By taking ${E}_{B}={\hslash }^{2}/m{a}^{2}$ as energy unit (then $\hslash {\omega }_{R}={\bar{\omega }}_{R}{E}_{B}$) and defining the *diluteness parameter* $\bar{n}=n{a}^{3}$, up to the linear term in ${\bar{\omega }}_{R}$, from equation (12) we obtain the scaled energy density of the mixture components with constant densities:

14 $$\begin{array}{l}\frac{ {\mathcal E} }{{E}_{B}/{a}^{3}}=\pi \mathrm{(1}+\varepsilon ){\bar{n}}^{2}-{\bar{\omega }}_{R}\bar{n}+\frac{8}{15{\pi }^{2}}{\mathrm{[2}\pi \bar{n}\mathrm{(1}+\varepsilon )]}^{\mathrm{5/2}}+\frac{8}{15{\pi }^{2}}{\mathrm{[2}\pi \bar{n}\mathrm{(1}-\varepsilon )]}^{\mathrm{5/2}}+\frac{14}{3{\pi }^{2}}{\bar{\omega }}_{R}{\mathrm{[2}\pi \bar{n}\mathrm{(1}-\varepsilon )]}^{\mathrm{3/2}}\mathrm{.}\end{array}$$

Notice that for ${\bar{\omega }}_{R}\mathrm{=0}$ one recover the Larsen’s zero-temperature equation of state^26^. From equation (14) one finds that for $|\varepsilon \mathrm{| > 1}$ the uniform configuration is not stable. If $\varepsilon \, > \,1$, at the mean field level, one expects phase separation or population imbalance^39^. Instead, if $\varepsilon  < -1$ the term proportional to ${\mathrm{[(1}+\varepsilon )\bar{n}]}^{\mathrm{5/2}}$ becomes imaginary. This imaginary term does not induce dynamical instability, but only dissipation. As for other sources of losses (for instance three-body recombination), to study short-time dynamics this dissipative term can be neglected if $\bar{n}$ is not too large. The resulting *real* energy density displays a characteristic ${\bar{n}}^{\mathrm{5/2}}$ dependence which competes with the negative mean-field contribution, opening the door to the possibility of observing a droplet phase for finite systems. This stabilization mechanism based on quantum fluctuations has been proposed for the first time in two-component mixtures without Rabi coupling^27,28^ and recently applied to dipolar condensates^32–34^. For $\varepsilon  < -1$ the equilibrium density is obtained upon the minimization of the energy density in equation (14) with respect to $\bar{n}$ neglecting the imaginary term:

15 $${\bar{n}}_{\pm }={(\frac{5\sqrt{\pi }\mathrm{|1}+\varepsilon |}{32\sqrt{2}{\mathrm{(1}+|\varepsilon |)}^{\mathrm{5/2}}}[1\pm \sqrt{1-\frac{1792{\bar{\omega }}_{R}}{15{\pi }^{2}}\frac{{\mathrm{(1}+|\varepsilon |)}^{4}}{\mathrm{|1}+\varepsilon {|}^{2}}}])}^{2}\mathrm{.}$$

The solution ${\bar{n}}_{-}$ is a local maximum, while the equilibrium value is given by ${\bar{n}}_{+}$ which is a local minimum of the energy per particle. Moreover to obtain a real solution, Rabi frequency is limited by: ${\bar{\omega }}_{R} < \frac{15{\pi }^{2}}{1792}\frac{\mathrm{|1}+\varepsilon {|}^{2}}{{\mathrm{(1}+|\varepsilon |)}^{4}}$. For larger ${\bar{\omega }}_{R}$ there is only the absolute minimum with zero energy at $\bar{n}=0$.

### Droplet phase

**Figure 2:**
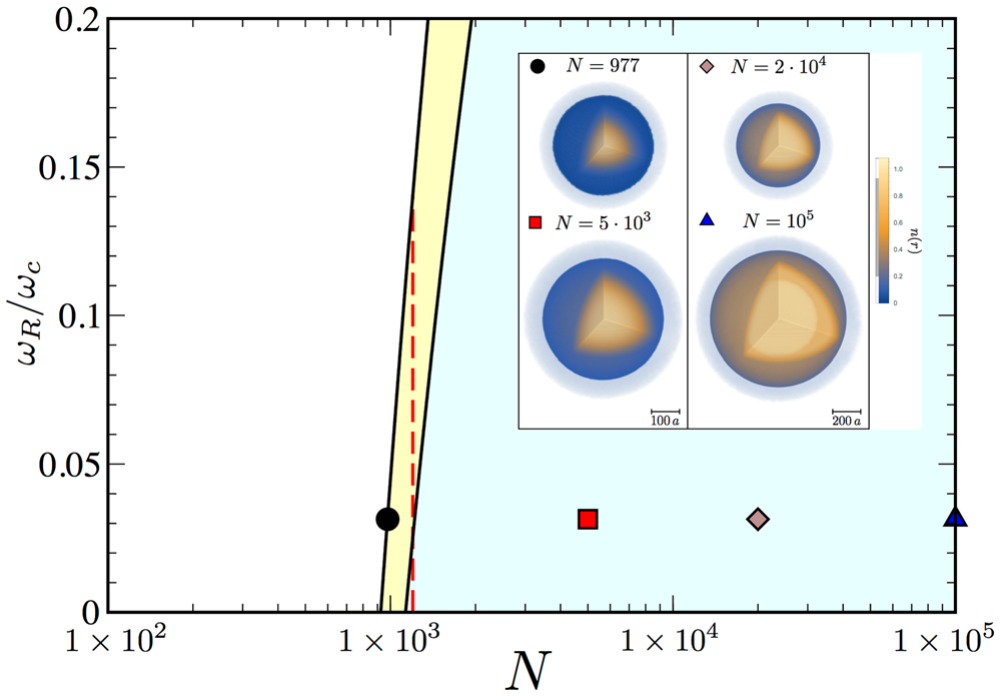
Stability diagram of the droplet phase. We identify the phases of a Rabi-coupled Bose mixture with equal number of particles upon the minimization of the energy functional $\bar{U}({\tilde{\sigma }}_{1},{\tilde{\sigma }}_{2},{\tilde{\sigma }}_{3})$ of equation (31). We observe three phases: a stable droplet-phase region (light green) of spherical self-bound droplets, a metastable droplet phase (yellow) where the energy of the droplet is positive and larger than a uniform background with vanishing density, and an unstable (white region) for small particle number *N* or high Rabi coupling $\omega$ where droplet evaporate. Here we consider $\mathrm{|1}+\varepsilon |=0.5$ which corresponds to ${\omega }_{c}\simeq 31.8\,$ kHz. In the inset we plot the three dimensional density profile $n(r)$ of droplets from the numerical solution of the Gross-Pitaevskii equation for different particle numbers at ${\omega }_{R}/2\pi =1$ kHz, from the metastable region $N=977$ and gaussian density limit $N=5\cdot {10}^{3}$ to the Thomas-Fermi regime $N=2\cdot {10}^{4}$ and $N={10}^{5}$ where system density is roughly constant up to a critical droplet radius. Moving along the vertical axis, increasing the Rabi coupling, droplets become metastable and finally unstable. Red dashed line refers to a system of $N=1200$ particles (see Methods).

For a finite system of $N$ of particles we define a space-time dependent complex field $\varphi ({\bf{r}},t)$ such that $n({\rm{r}},t)=|\varphi ({\bf{r}},t{)|}^{2}$ is the space-time dependent local number density, and clearly $N=\int {d}^{3}{\bf{r}}n({\bf{r}},t)$. The dynamics of $\varphi ({\bf{r}},t)$ is driven by the following real-time effective action

16 $${S}_{{\rm{eff}}}[{\varphi }^{\ast },\varphi ]=\int dt\,{d}^{3}{\bf{r}}[i\hslash {\varphi }^{\ast }{\partial }_{t}\varphi -\frac{{\hslash }^{2}|\nabla \varphi {|}^{2}}{2m}-{ {\mathcal E} }_{ND}(|\varphi {|}^{2})],$$

where ${ {\mathcal E} }_{ND}$ is obtained from equation (14) neglecting the imaginary term proportional to ${\mathrm{[(1}+\varepsilon )\bar{n}]}^{\mathrm{5/2}}$. In the inset of Fig. 2 we plot the density profile of the stationary solution obtained by numerically solving with imaginary time-evolution the Gross-Pitaevskii equation associated to equation (16) varying the number of particles for ${\omega }_{R}/2\pi =1$ kHz. The solution indeed corresponds to a self-bound spherical droplet whose radial width increases by increasing the number of atoms. For a very large number of atoms, the plateau of the density profile approaches the thermodynamic density given by equation (15). Instead, for a small number of atoms the self-bound droplet does not exist.

One can model the droplet by using a Gaussian wavefunction

17 $$\tilde{\varphi }(\tilde{{\bf{r}}},\tilde{t})=\frac{\sqrt{\tilde{N}}}{{\pi }^{\mathrm{3/4}}\sqrt{{\tilde{\sigma }}_{1}{\tilde{\sigma }}_{2}{\tilde{\sigma }}_{3}}}\prod _{i=1}^{3}\exp [-\frac{{\tilde{x}}_{i}^{2}}{2{\tilde{\sigma }}_{i}^{2}}+i{\tilde{\beta }}_{i}{\tilde{x}}_{i}^{2}]$$

where ${\tilde{\sigma }}_{i}(t)$ and ${\tilde{\beta }}_{i}(t)$ are time-dependent variational parameters rescaled in units of *a*. Here we set ${\bf{r}}=a\tilde{{\bf{r}}}$ and $|\varphi |=\sqrt{n}|\tilde{\varphi }|$. The normalization condition then becomes $\int {d}^{3}\tilde{{\bf{r}}}|\tilde{\varphi }{|}^{2}=\tilde{N}$ where the particle number is $N=\tilde{N}\bar{n}$. By inserting equation (17) in the rescaled version of equation (16), one gets six Euler-Lagrange equations for the parameters ${\{{\tilde{\sigma }}_{i},{\tilde{\beta }}_{i}\}}_{i}$, i.e ${\tilde{\beta }}_{i}={\dot{\tilde{\sigma }}}_{i}/2{\tilde{\sigma }}_{i}$ and ${\ddot{\tilde{\sigma }}}_{i}=-\partial \bar{U}({\tilde{\sigma }}_{1},{\tilde{\sigma }}_{2},{\tilde{\sigma }}_{3})/\partial {\tilde{\sigma }}_{i}$. $\bar{U}({\tilde{\sigma }}_{1},{\tilde{\sigma }}_{2},{\tilde{\sigma }}_{3})$ ^46^ is a variational energy functional which is function of the width of the droplet only (see Methods). The variational stability diagram of the droplet phase is illustrated in Fig. 2. Upon increasing the atom number droplets stabilize. For small particle numbers we find a metastable region where $\bar{U}({\tilde{\sigma }}_{1},{\tilde{\sigma }}_{2},{\tilde{\sigma }}_{3})$ has a local minimum with positive energy, the global minimum corresponding to zero energy for a dispersed gas with zero density. Interestingly, tuning the Rabi coupling to large values, as shown with the red dashed line for $N=1200$ particles in Fig. 2, we move into the unstable phase. Therefore, differently from dipolar gases^32^ or bosonic mixtures with attractive inter-species interactions^27^, where transition to the instability is driven by interactions, here, a direct coupling between the two components serves as an additional tunable knob to cross from a stable into an unstable phase.

The low-energy collective excitations of the self-bound droplet are investigated by solving the eigenvalues problem for the Hessian matrix of effective potential energy in equation (31). From the form of the variational ansatz we naturally describe the monopole (breathing) mode of frequency ${\omega }_{M}$ and the quadrupole mode of frequency ${\omega }_{Q}$.

**Figure 3:**
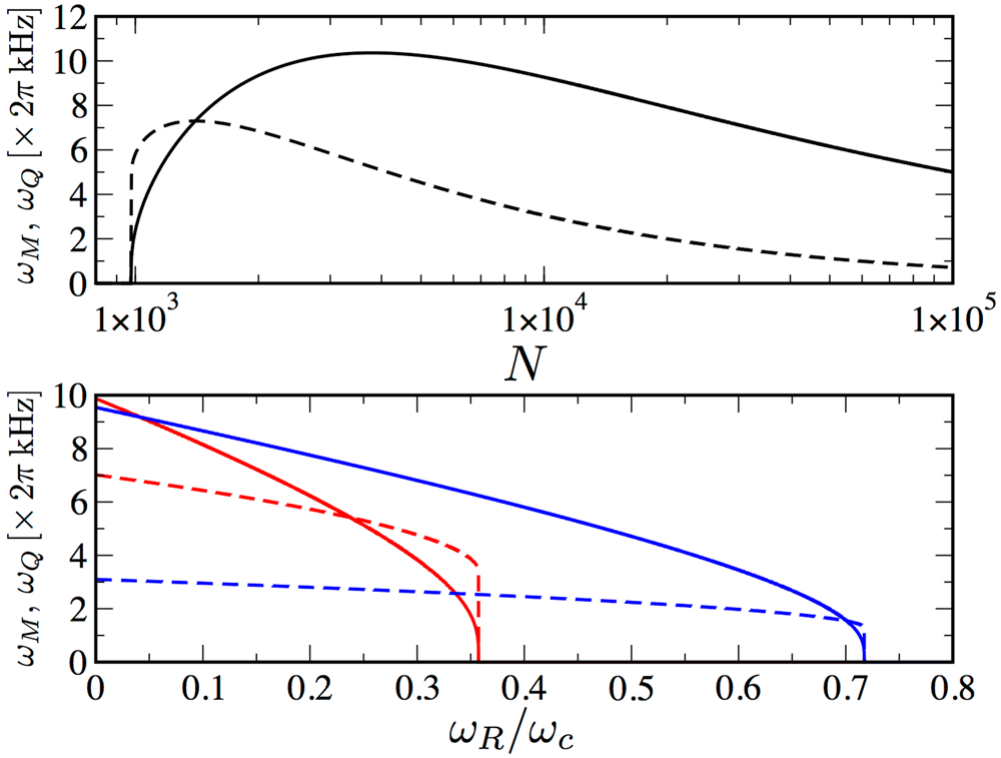
Collective excitations of droplets. Monopole (breathing) mode frequency ${\omega }_{M}$ (solid) and quadrupole mode frequency ${\omega }_{Q}$ (dashed) with $\mathrm{|1}+\varepsilon |=0.5$. Upper panel: frequencies as a function of particle number and ${\omega }_{R}/2\pi =1\,{\rm{kHz}}$. Below $N\simeq 977$ the droplet becomes unstable. Lower panel: frequencies as a function of Rabi coupling for $N=2\cdot {10}^{3}$ (red), and $N={10}^{5}$ (blue). The critical Rabi frequency occurs at ${\omega }_{c}/2\pi =31.8$ kHz.

The upper panel of Fig. 3 displays monopole and quadrupole frequencies as a function of the number *N* of atoms in the droplet, fixing Rabi coupling and scattering lengths. The lower panel of Fig. 3 reports the collective frequencies as a function of the Rabi coupling and two different values of *N*. Both frequencies go to zero at the Rabi coupling above which the droplet evaporates.

The experimental observation of a droplet phase with Rabi coupled internal states is within experimental reach. A promising candidate is a gas of ^39^K atoms loaded in hyperfine states $|F=\mathrm{1,}{m}_{F}=0\rangle$ and $|F=\mathrm{1,}{m}_{F}=-1\rangle$. The narrow Feshbach resonance at $B\simeq 54.5$ G for collisions between atoms in $\mathrm{|1,0}\rangle$, allows to tune intra-component scattering length to equal values to the intra-component one for the state $\mathrm{|1,}-1\rangle$, then ${a}_{1}={a}_{2}\simeq 40$ ${a}_{0}$, where ${a}_{0}$ is the Bohr radius^47,48^. The corresponding inter-component scattering length is ${a}_{12}\simeq -60{a}_{0}$, which gives $\varepsilon \simeq -1.5$. For a Rabi coupling frequencies of the order of ${\omega }_{R}/2\pi =1$ kHz^49^ and $N={10}^{5}$ particles, we predict a droplet with a FWHM $\simeq 1.45$ $\mu$ m.

## Discussion

We derived the beyond-mean-field grand potential of a Rabi-coupled bosonic mixture within the formalism of functional integration, and performing regularization of divergent Gaussian fluctuations. In the small Rabi-coupling regime we also obtained an analytical expression for the internal energy of the system. In the case of attractive inter-particle scattering length we have shown how the Gaussian terms of the internal energy help to stabilize the system against the collapse and that, for a finite number of atoms, a self-bound droplet is produced. Rabi coupling works as an additional tool to tune the stability properties of the droplet, inducing an energetic instability for large inter-component couplings. The evaporation of the droplet is also signaled by both the breathing and quadruple modes which vanish at a critical Rabi coupling. Notably, our predictions provide a benchmark for experimental observations of Rabi-coupled self-bound droplets in current experiments.

## Methods

### Mean-field phase diagram

Our description of mean-field phase diagram starts from the mean-field free-energy density, where we identified ${v}_{a}^{2}={n}_{a}$, ${v}_{b}^{2}={n}_{b}$ and consequently $n={v}_{a}^{2}+{v}_{b}^{2}$

18 $$\frac{{{\rm{\Omega }}}_{0}}{{L}^{3}}=-\mu n+\frac{1}{2}g({n}_{a}^{2}+{n}_{b}^{2})+{g}_{ab}{n}_{a}{n}_{b}-2\hslash {\omega }_{R}\sqrt{{n}_{a}{n}_{b}}\mathrm{.}$$

The equilibrium configuration has to stationarize the energy density, namely

19 $$[g-{g}_{ab}+\frac{\hslash {\omega }_{R}}{\sqrt{{n}_{a}{n}_{b}}}]({n}_{a}-{n}_{b})=0$$

It is possible to characterize the equilibrium configuration by means of the population imbalance between the species ${\rm{\Delta }}={n}_{a}-{n}_{b}$. In terms of ${\rm{\Delta }}$, the solutions of equation (19) are given by^39^

20 $$\begin{array}{ll}{\rm{Symmetric}}\,\,{\rm{Ground}}\,\,{\rm{State}}: & {\rm{\Delta }}=0\\ {\rm{Polarized}}\,\,{\rm{Ground}}\,\,{\rm{State}}: & {\rm{\Delta }}=\pm n\sqrt{1-{[\frac{2\hslash {\omega }_{R}}{n(g-{g}_{ab})}]}^{2}}\end{array}.$$

In order to clarify the stability of these equilibrium points, one has to compute the determinant of the free-energy Hessian matrix (we assume an intra-species repulsion). Over the symmetric ground state, one finds

21 $${\rm{\det }}[\frac{1}{{L}^{3}}\frac{{\partial }^{2}{{\rm{\Omega }}}_{0}}{\partial {n}_{a}\partial {n}_{b}}]=(g+{g}_{ab})(g-{g}_{ab}+\frac{2\hslash {\omega }_{R}}{n})$$

and by imposing it to be positive, the following stability condition^39^

22 $${g}_{ab} < g+2\frac{\hslash {\omega }_{R}}{n}\mathrm{.}$$

In the symmetric ground-state, the density in terms of $\mu$ reads $n/2=(\mu +\hslash {\omega }_{R})/(g+{g}_{ab})$, so we easily derive equation (7). In the polarized ground-state, since $n=\mu /g$, the normalized imbalance equals

23 $$\frac{{\rm{\Delta }}}{n}=\pm \sqrt{1-{[\frac{2g{\omega }_{R}}{\mu (g-{g}_{ab})}]}^{2}},$$

leading to the stability condition

24 $${g}_{ab} > \frac{2\hslash {\omega }_{R}}{\mu }g\mathrm{.}$$

The results of stability analysis of the stationary points of mean-field free energy are summarized in Fig. 1 (top panel).

### Quantum fluctuations and equation of state

The inverse propagator introduced in equation (8) is defined by:

25 $$-\hslash {\mathbb{M}}({\bf{q}},{\omega }_{n})=(\begin{array}{ll}-\hslash {{\mathscr{G}}}^{-1}({\bf{q}},{\omega }_{n}) & \hslash {\Sigma }_{12}\\ \hslash {\Sigma }_{12} & -\hslash {{\mathscr{G}}}^{-1}({\bf{q}},{\omega }_{n})\end{array})$$

(see e.g. Refs^35,37^), where:

26 $$-\hslash {{\mathscr{G}}}^{-1}=(\begin{array}{ll}-i\hslash {\omega }_{n}+{h}_{q} & gv/\sqrt{2}\\ gv/\sqrt{2} & i\hslash {\omega }_{n}+{h}_{q}\end{array}),$$

is the single component inverse propagator, with ${h}_{q}={\varepsilon }_{q}+g{v}^{2}+{g}_{12}{v}^{2}/2-\mu$ and ${\varepsilon }_{q}={\hslash }^{2}{q}^{2}/2m$. The off-diagonal blocks of ${\mathbb{M}}({\bf{q}},{\omega }_{n})$ are given by

27 $$\hslash {\Sigma }_{12}=(\begin{array}{ll}{g}_{12}{v}^{2}/2-\hslash {\omega }_{R} & {g}_{12}{v}^{2}/2\\ {g}_{12}{v}^{2}/2 & {g}_{12}{v}^{2}/2-\hslash {\omega }_{R}\end{array}).$$

describes the inter-component coupling.

### Convergence-factor regularization technique

Among the available methods to regularize the zero-point energy of bosonic excitations^43^, we employ the convergence-factor technique. It consists in adding a factor ${e}^{i{\omega }_{n}{0}^{+}}$ before performing the Matsubara summation contained in equation (8); this notation has to be intended as a limit procedure, i.e ${lim}_{\delta \to {0}^{+}}{e}^{i{\omega }_{n}\delta }$. As an example, we consider single-state bosons^37^ with energy $\varepsilon$. The partition function is given by ${\mathscr{Z}}={\rm{T}}{\rm{r}}[\exp (-\beta (\varepsilon -\mu ){\hat{\psi }}^{\dagger }\hat{\psi })]$, and in the path-integral framework, one formally writes

28 $${\mathscr{Z}}=\int {\mathscr{D}}[\psi ,{\psi }^{\ast }]\exp \{-\frac{1}{\hslash }{\int }_{0}^{\hslash \beta }d\tau \,{\psi }^{\ast }(\tau )(\hslash {{\rm{\partial }}}_{\tau }+\varepsilon -\mu )\psi (\tau )\}.$$

Since the path-integral relies on a time-axis discretization, this notation introduces an ambiguity^37^. We are not specifying on which time slice the field ${\psi }^{\ast }(\tau )$ (corresponding to the operator ${\hat{\psi }}^{\dagger }$) acts. If $\psi (\tau )$ acts on the time slice ${\tau }_{i}$, then we can choose that $\psi (\tau +\delta )$ acts on the ${\tau }_{i+1}$ one, and $\delta \to {0}^{+}$ is needed to specify this prescription. In the Fourier space, it corresponds to the appearance of the convergence factor presented at the beginning of this subsection. If one instead chooses the opposite time-ordering, by taking the limit $\delta \to {0}^{-}$, one can then verify that the corresponding partition functions differ only in a ${e}^{-\beta (\varepsilon -\mu )}$. factor. Then, from a partition function as the one in equation (28), with fields computed at the same time, we need to add a term such as $\frac{1}{2}(\varepsilon -\mu )$ in the grand potential to take this fact into account. This justifies the first two counterterm appearing in equation (13).

However, this equal-time prescription does not completely remove the ultraviolet divergences in the zero-point energy of bosonic excitations. The remaining ones are due to the presence of a gapped spectrum branch and to the zero-range approximation for the interaction potential. The grand potential of our system with Gaussian contribution is given by

29 $${\rm{\Omega }}={{\rm{\Omega }}}_{0}(\mu )+\frac{1}{2\beta }\sum _{{\bf{q}},{\omega }_{n}}\mathrm{log}\,{\rm{\det }}\,{\mathbb{M}}({\bf{q}},i{\omega }_{n}),$$

where ${\mathbb{M}}({\bf{q}},i{\omega }_{n})$ is defined in equation (25). Gapped excitations induce a branch cut in the complex logarithm on the real *q*-axis at higher energy, corresponding to the single-particle continuum of states. This divergent contribution is removed as shown by Diener *et al*.^45^, giving rise to the third counteterm in equation (13). Finally, the fourth counterterm arises by making use of standard scattering theory at the second order^44^, namely

30 $$\frac{m}{4\pi {\hslash }^{2}{a}_{s}}=\frac{1}{g}+\int \frac{{d}^{3}{\bf{q}}}{{\mathrm{(2}\pi )}^{3}}\frac{m}{{\hslash }^{2}{q}^{2}}\mathrm{.}$$

Indeed, one of the divergences encountered integrating the zero-point energy is due to the delta-shaped potential^37^. Its Fourier transform is constant for all momenta, while a reasonable interaction potential should fall at least as $\mathrm{1/}{q}^{2}$, giving back a finite contribution.

### Variational and numerical analysis

The equation for ${\tilde{\sigma }}_{i}$ is the classical equation of motion for a particle of coordinates $\tilde{{\boldsymbol{\sigma }}}={({\tilde{\sigma }}_{1},{\tilde{\sigma }}_{2},{\tilde{\sigma }}_{3})}^{T}$ moving in an effective potential given by the derivative of the potential energy per particle:

31 $$\begin{array}{ll}\bar{U}(\tilde{\sigma }) & =\frac{1}{2}\sum _{i\mathrm{=1}}^{3}\frac{1}{2{\tilde{\sigma }}_{i}^{2}}-\frac{\mathrm{|1}+\varepsilon |N}{2\sqrt{2\pi }({\tilde{\sigma }}_{1}{\tilde{\sigma }}_{2}{\tilde{\sigma }}_{3})}+\alpha \frac{{\mathrm{(1}+|\varepsilon |)}^{\mathrm{5/2}}{N}^{\mathrm{3/2}}}{{({\tilde{\sigma }}_{1}{\tilde{\sigma }}_{2}{\tilde{\sigma }}_{3})}^{\mathrm{3/2}}}+\gamma \frac{{\mathrm{(1}+|\varepsilon |)}^{\mathrm{3/2}}{\bar{\omega }}_{R}{N}^{\mathrm{1/2}}}{{({\tilde{\sigma }}_{1}{\tilde{\sigma }}_{2}{\tilde{\sigma }}_{3})}^{\mathrm{1/2}}}\end{array}$$

where $$\alpha =\frac{128}{75\sqrt{5}{\pi }^{\mathrm{7/4}}}$$ and $$\gamma =\frac{112}{9\sqrt{3}{\pi }^{\mathrm{5/4}}}$$.

The energy per particle of the ground state is simply ${\bar{E}}_{{\rm{gs}}}/N=\bar{U}({\tilde{\sigma }}_{m})$ where ${\tilde{\sigma }}_{m}$ is the minimum of the effective potential energy. In absence of an external trapping, the system preserves its spherical symmetry, i.e. the critical point of the effective potential in equation (31) is for ${\tilde{\sigma }}_{m1}={\tilde{\sigma }}_{m2}={\tilde{\sigma }}_{m3}$. The time dependence of ${\tilde{\beta }}_{i}$ is completely determined by the one of ${\tilde{\sigma }}_{i}$ ^46^.

**Figure 4:**
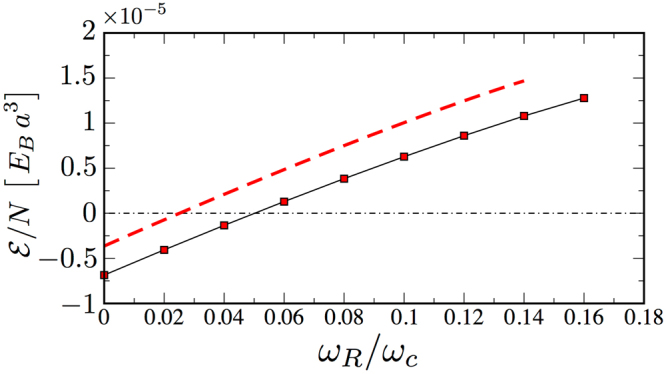
Energetic instability of droplets. Energy per particle of a system of $N=1200$ particles as a function of the Rabi coupling along the vertical line of Fig. 2. Red dashed line: Variational energy from equation (31). Squared dots: Energy per particle from the numerical solution of the Gross-Pitaevskii equation. Increasing the Rabi coupling to values larger than $\omega \simeq 0.16\,{\omega }_{c}$ the *metastable* droplet evaporates.

Figure 4 shows the energy per particle of the self-bound droplet: the numerical approach relying on imaginary-time evolution is in reasonable agreement with the variational one based on equation (17). Remarkably, above a critical Rabi frequency the internal energy of the droplet becomes positive, signaling that the droplet goes in a metastable configuration. Moreover, at a a slightly larger critical Rabi frequency the droplet evaporates.

### Data availability

Data are available upon request. Requests should be addressed to either author.
